# Simultaneous targeting of mitochondrial Kv1.3 and lysosomal acid sphingomyelinase amplifies killing of pancreatic ductal adenocarcinoma cells in vitro and in vivo

**DOI:** 10.1007/s00109-023-02290-y

**Published:** 2023-02-15

**Authors:** Sameer H. Patel, Magdalena Bachmann, Stephanie Kadow, Gregory C. Wilson, Mostafa M. L. Abdel-Salam, Kui Xu, Simone Keitsch, Matthias Soddemann, Barbara Wilker, Katrin Anne Becker, Alexander Carpinteiro, Syed A. Ahmad, Ildiko Szabo, Erich Gulbins

**Affiliations:** 1 Department of Surgery, University of Cincinnati College of Medicine, Cincinnati, OH USA; 2 Department of Biologyand , CNR Institute of Neurosciences, University of Padua, Padua, Italy; 3 Institute of Molecular Biology, University Hospital Essen, University of Duisburg-Essen, Hufelandstrasse 55, 45122 Essen, Germany

**Keywords:** Pancreas cancer, Acid sphingomyelinase, Sphingolipids, Lysosomes, Kv1.3, Mitochondria

## Abstract

**Abstract:**

Pancreas ductal adenocarcinoma (PDAC) remains a malignant tumor with very poor prognosis and low 5-year overall survival. Here, we aimed to simultaneously target mitochondria and lysosomes as a new treatment paradigm of malignant pancreas cancer in vitro and in vivo. We demonstrate that the clinically used sphingosine analog FTY-720 together with PAPTP, an inhibitor of mitochondrial Kv1.3, induce death of pancreas cancer cells in vitro and in vivo. The combination of both drugs results in a marked inhibition of the acid sphingomyelinase and accumulation of cellular sphingomyelin in vitro and in vivo in orthotopic and flank pancreas cancers. Mechanistically, PAPTP and FTY-720 cause a disruption of both mitochondria and lysosomes, an alteration of mitochondrial bioenergetics and accumulation of cytoplasmic Ca^2+^, events that collectively mediate cell death. Our findings point to an unexpected cross-talk between lysosomes and mitochondria mediated by sphingolipid metabolism. We show that the combination of PAPTP and FTY-720 induces massive death of pancreas cancer cells, thereby leading to a substantially delayed and reduced PDAC growth in vivo.

**Key messages:**

- FTY-720 inhibits acid sphingomyelinase in pancreas cancer cells (PDAC).
- FTY-720 induces sphingomyelin accumulation and lysosomal dysfunction.
- The mitochondrial Kv1.3 inhibitor PAPTP disrupts mitochondrial functions.
- PAPTP and FTY-720 synergistically kill PDAC in vitro.
- The combination of FTY-720 and PAPTP greatly delays PDAC growth in vivo.

## Introduction

Pancreas ductal adenocarcinoma (PDAC) is a challenging disease to treat and is the third leading cause of cancer related mortality [1]. Although surgery is the only curative treatment for patients with PDAC, only 15–20% of patients are candidates. Even in this group, 5-year overall survival is less than 20%. Cytotoxic chemotherapies are used along with surgery, but are plagued with inevitable cancer progression and toxicities with use of multi-drug regimens [2]. Immunotherapy has increasingly been efficacious in many gastrointestinal malignancies but has shown minimal benefit in PDAC [3]. Therefore, novel treatment strategies that can work outside of the mechanisms of traditional cytotoxic chemotherapies to target the tumor are desperately needed.

Ion channels are transmembrane proteins that form aqueous pores driving ions through membranes in favor of an electrochemical gradient.

We have previously shown a dual, functional expression of Kv1.3 in the plasma membrane and the inner mitochondrial membrane (IMM) of lymphocytes [4–10]. We have demonstrated that direct inhibition of mtKv1.3, either by Bax and Bak or by membrane-permeant small-molecule inhibitors of Kv1.3, i.e. clofazimine, Psora-4 and PAP-1, induces cell death by triggering a transient mitochondrial hyperpolarization, release of mitochondrial reactive oxygen species (ROS), release of cytochrome c and opening of the permeability transition pore (PTP) leading to strong dissipation of the membrane potential of the inner mitochondrial membrane [10–14].

We also introduced novel inhibitors of Kv1.3, named PAPTP and PCARBTP, that specifically target mitochondria [8, 12, 13]. These drugs drastically reduced melanoma and pancreatic ductal adenocarcinoma (PDAC) growth in orthotopic models, without affecting healthy, non-malignant cells [8, 12, 13]. PCARBTP and PAPTP directly and efficiently affected mitochondrial function; induced mitochondrial swelling, fragmentation of the mitochondrial network, dissipation of Δφm, formation of ROS, and release of cytochrome c; reduced maximal respiration; and finally abrogated ATP production by mitochondria [8].

Sphingolipids are a class of lipids that share an amino alcohol (sphingoid base) backbone. Acid sphingomyelinase, the enzyme converting sphingomyelin to ceramide, is present in lysosomes, but since these compartments are constantly recycling to the plasma membrane, it can be also found on the cell surface and secreted from cells [15, 16]. In addition, the acid sphingomyelinase is present in the inter-membrane space of mitochondria [17]. Genetic deficiency of the acid sphingomyelinase results in massive accumulation of sphingomyelin and a lysosomal storage disease, Niemann-Pick disease Type A/B. Pharmacological inhibition of the acid sphingomyelinase results in much less pronounced increase of sphingomyelin in lysosomes, but has been shown to contribute to lysosomal permeabilization, the release of lysosomal enzymes such as cathepsins into the cytoplasm and finally cell death [18, 19]. This process seems to be relatively specific for malignant tumor cells, because lysosomes in malignant cells are altered in composition, stability, and pH and seem to be more fragile than lysosomes in benign cells [20].

Here, we investigated the effect of a sphingosine analog, FTY-720, and an inhibitor of mitochondrial Kv1.3 on pancreas cancer cells. FTY-720 is currently used in the clinic against multiple sclerosis [21], since phosphorylated FTY-720 acts as an inhibitor on sphingosine 1-phosphate receptors and modifies the function of auto-immune cells [21]. However, FTY-720 has been also shown to inhibit the acid sphingomyelinase and may interfere with the consumption of sphingomyelin and the generation of ceramide and thereby induces lysosomal membrane permeabilization [22–24].

Here we hypothesized that simultaneous disruption of both mitochondrial and lysosomal functions substantially enhances cell death of pancreas cancer cells. We demonstrate that the combination of PAPTP and FTY-720 induces massive cell death in pancreas cancer cells in vitro and also acts against the tumor in vivo. Mechanistically, we demonstrate that the combination of PAPTP and FTY720 targets both mitochondria and lysosomes and thereby mediates rapid and massive cell death of PDAC cells.

## Methods

### Mice and treatments

#### Ethics approval

All animal experiments were approved by the University of Cincinnati Ethic Committee and the Institutional Animal Care and Use Committee.

All experiments were performed according to the FELASA regulations and we also followed the ARRIVE guidelines. Eight-week-old, wild type C57BL/6 J mice were employed. Mice were anesthetized using 120 mg/kg ketamine plus 20 mg/kg xylazine, a left subcostal incision was made just below rib cage and the pancreas was identified. Orthotopic injection was performed as described by [35]. In detail, the tumor cell suspension was achieved by resuspending 1 × 10^6^ KC cells in 25 μL PBS with 25 μL Matrigel. The tumor suspension was slowly injected into the pancreas and the needle left in place for 60 s to allow the Matrigel to set. After ensuring hemostasis, the abdomen was closed in 2 layers using 3–0 silk suture. Flank tumors were induced by injecting 50,000 KC cells in 50 μL HEPES/Saline (H/S; 132 mM NaCl, 20 mM HEPES [pH 7.4], 5 mM KCl, 1 mM CaCl_2_, 0.7 mM MgCl_2_, 0.8 mM MgSO_4_) subcutaneously into the right flank. Tumor-injected mice were randomly divided into experimental groups.

FTY-720, PAPTP and the combination of both were injected intravenously at a dose of 10 mg/kg for FTY-720 [36] and 3 mg/kg for PAPTP [8] in a total volume of 125 μL DMSO and 0.9% NaCl (1:4, v/v). Controls were injected with the solvent only.

Tumor-bearing mice were injected at day 7 for biochemical studies. Tumors were removed 15, 30, or 60 min after drug injection. To determine the effect of the drugs on tumor size, the mice were injected on day 2, 4, 6, and 8 after tumor initiation with PAPTP + FTY-720 or solvent control. Mice were sacrificed and tumor size was calculated when the tumors became clearly visible, but still had no impact on health of the mice.

### Cells and cellular stimulations

KC or KPC tumor cells were generated from PDAC tumors containing Kras^LSL.*G12D/*+^;Pdx-1-Cre (KC), *Kras*^*LSL−G12D/*+^, *Trp53*^*loxP/*+^, *Pdx1-Cre* (KPC) mice in C57BL/6 mice background (Jackson Laboratories). Rodent pathogen status of the tumor cell lines were verified by Charles River Laboratory diagnosis services. Cells were cultured in modified Eagle medium (MEM) supplemented with 10 mM HEPES (pH 7.4; Carl Roth GmbH, Karlsruhe, Germany), 2 mM l-glutamine, 1 mM sodium pyruvate, 100 μM nonessential amino acids, 100 U/mL penicillin, 100 μg/mL streptomycin, and 10% fetal calf serum. For stimulation, cells were washed twice in MEM supplemented with 10 mM HEPES. Cells were then stimulated with 0.1–1 μM PAPTP, 10 μM FTY-720 or PAPTP (0.1–1 μM) + FTY-720 (10 μM) for the indicated times in MEM supplemented with 10 mM HEPES. Drugs were diluted in DMSO and then in MEM/10 mM HEPES. The final concentration of DMSO was 0.5%. To test the effects of BAPTA on cell death, cells were incubated with 5 μM BAPTA in addition to PAPTP + FTY-720.

### Genetic downregulation of acid sphingomyelinase, sphingosine kinase 1 or sphingosine kinase 2

Expression of the acid sphingomyelinase, sphingosine kinase 1 or sphingosine kinase 2 in KC cells was down-regulated by transfection with commercial shRNA targeting the acid sphingomyelinase (Santa Cruz Inc., # sc-41651), sphingosine kinase 1 (Santa Cruz Inc., # sc-45446) or sphingosine kinase 2 (Santa Cruz Inc., # sc-39225-SH). Control cells were transfected with an irrelevant shRNA (Santa Cruz Inc. # sc-108060). Cells were stably transfected by electroporation at 400 V with 5 pulses, 3 ms each, using a BTX electroporator. Transfected cells were selected with puromycin (3.5 μg/mL). Bulk cultures were employed in the present experiments. Puromycin was removed from the cultures 7 days prior to any experiment. Downregulation of the acid sphingomyelinase was confirmed by measuring the activity of the enzyme in transfected, control-transfected, and untransfected cells. Downregulation of sphingosine kinase 1 and 2 was confirmed by western blotting. We used stably transfected cells, which were selected with puromycin. Thus, all cells expressed the shRNA construct. We used cultures in which expression of the acid sphingomyelinase was only reduced by approximately 50% to prevent cellular changes that are associated with a complete deficiency of the acid sphingomyelinase, in particular a very strong accumulation of sphingomyelin prior to treatment.

### Cell death and FITC-Annexin V binding

KC or KPC cells were treated as indicated, trypsinized, washed in H/S, stained for 15 min with FITC-Annexin V (Roche), and analyzed by flow cytometry on a FACS-Calibur. Controls were permeabilized for 5 min with 0.1% Triton X-100 at room temperature before incubation with FITC-Annexin V.

Cell death was confirmed by Trypan Blue staining and counting of dead cells.

### Soft agar colony formation

Soft agar colony formation assay has been performed as described in [37]. Briefly, 600 μL of agar (1% dissolved in DMEM without Phenol Red and FCS) was layered and kept solidifying for 4 h at 4 °C in 12 well-plate. 10,000 KC cells (in 600 μL of agar, 0.6% dissolved in phenol-red DMEM medium with 2% FCS) were then seeded. After 45 min, 1 mL of DMEM medium + 4% FBS was added and samples were incubated for 24 h. On the following day, cells were treated with PAPTP, FTY720, or both for 48 h, and then the medium was removed and replaced with fresh medium twice a week. Colonies were cultured for 3 weeks and stained with 0.005% Crystal violet for 2 h. Images were captured with Leica MZ16 F microscope, and the numbers and the area of the colonies were determined using ImageJ software.

### Electron microscopy studies

For transmission electron microscope images, 15 000 KC cells were seeded in 24-w plates two days prior to treatment. Cells were treated with 0.1% DMSO (vehicle control), 1 μM PAPTP, 10 μm FTY-720 or a combination of both for 30 or 180 min as indicated in the figures. After treatment, cells were fixed for transmission electron microscopy in a 2.5% (v/v) glutaraldehyde solution in 100 mM sodium cacodylate, pH 7.2, at 4 °C overnight. Following washing, post-fixation was performed in a 1% OsO4 solution in 100 mM sodium cacodylate, pH 7.2, at 4 °C. Sections were contrasted with a saturated uranyl acetate solution in 100% ethanol for 15 min, followed by incubation in a 1% (w/v) lead citrate solution in 100% ethanol for 7 min. Finally, the samples were observed with a Tecnai G2 Spirit transmission electron microscope (Fei electron microscopes) operating at 100 kV.

### Mitochondrial membrane potential and ROS production

4000 KC cells/well were seeded in glass bottom 96-well cell imaging plates in standard culture medium and incubated for 24 h. Then, cells were incubated with either 25 nM Tetramethylrhodamine (TMRM) (ThermoFisher) or 2.5 μM mitoSOX Red (ThermoFisher) together with 5 μg/ml Hoechst 33,342 (ThermoFisher) in HBSS for 20 min at 37 °C. The medium was then substituted with HBSS (supplemented with 5 nM TMRM in the case of membrane potential determinations) and cells were imaged for 10 min using the Operetta system (Perkin Elmer). After 10 min, treatment compounds at the indicated concentration were added and cells were imaged every 5 min for 40 min. 5 fields/well were analyzed. Analysis was performed using Harmony high-content analysis software. For the determination of ROS by FACS analysis, cells were treated for the indicated times, washed with PBS and harvested. After washing, the cells were resuspend in 500 μL of HBSS + MitoSOX (1:1000) and incubated for 30 min at 37 °C and following washes, analyzed with Flow cytometer.

### Seahorse

Cells were cultured on Extracellular Flux Cartridges for oxygen consumption rate (OCR) and extracellular acidification rate (ECAR) measurements (GE, # 103,723–100) exactly following the protocols provided by the supplier. Cells on the cartridges were washed and analyzed in a Seahorse XF HS Mini. To this end, the cells were equilibrated and warmed to 37 °C for approximately 15 min; PAPTP (1 μM), FTY-720 (10 μM), or the combination of the drugs were added; and the OCR and ECAR were determined online for 20 min. We then added oligomycin (3.75 μM), a blocker of H^+^-ATPase and continued to record for and addition 20 min. We then added rotenone (0.5 μM) and antimycin A (0.5 μM) to block complex I and III and recorded for an additional 20 min.

### Acid sphingomyelinase activity

Acid sphingomyelinase activity was determined in KC and KPC cells. To this end, cells were grown in 24-well plates and washed with H/S and cells were treated in MEM supplemented with 1% FCS and 2 mM glutamine with 0.1–1 μM PAPTP, 10 μM FTY-720 or PAPTP (0.1–1 μM) + FTY-720 (10 μM) for the indicated times. Stimulation was stopped by washing the cells once in ice-cold and cells were lysed in in 250 mM sodium acetate (pH 5.0) and 1% NP40 for 5 min. The lysates were removed from the plates and diluted to 0.2% NP40 in 250 mM sodium acetate (pH 5.0). If the tumors were grown in vivo (orthotopic pancreas or flank tumors), the tumors were removed, shock-frozen in liquid nitrogen (LN2), and homogenized and lysed by tip sonication in 0.2% NP40 in 250 mM sodium acetate (pH 5.0). The enzyme assay was initiated by addition of 0.05 μCi [^14^C]sphingomyelin (52 mCi/mmol; ARC) to the samples. [^14^C]sphingomyelin was dried for 10 min in a SpeedVac, resuspended in 250 mM sodium acetate (pH 5.0) and 0.1% NP40, and sonicated for 10 min in a bath sonicator prior to use. The samples were incubated for 30 min at 37 °C with shaking at 300 rpm and then extracted in 4 volumes of CHCl_3_:CH_3_OH (2:1, v/v). The samples were centrifuged, and an aliquot of the upper aqueous phase was scintillation-counted to determine the release of [^14^C]phosphorylcholine from [^14^C]sphingomyelin.

### Quantitative measurement of sphingomyelin

Sphingomyelin was determined by ELISA employing a kit from Assay Genie (HUES02806). Cells and tumors were lysed in 0.2% NP40 in H_2_O as above. Aliquots (100 μL) of the lysates were incubated on the plates pre-coated with anti-sphingomyelin antibodies for 90 min at 37 °C. Samples were aspirated and 100 μL of biotinylated anti-sphingomyelin detection antibody was added and incubated with the samples for 60 min at 37 °C. Plates were extensively washed 5 times and 100 μL of the HRP-streptavidin conjugate were added. Samples were incubated for 30 min at 37 °C, washed 5 times, and incubated with substrate reagent for 15 min at 37 °C, the reaction was stopped, and OD values were read at 450 nm. Values were determined according to a standard curve with known concentrations of sphingomyelin.

### Quantification of ceramide

In vivo tumors or KC or KPC cells were treated as described above, lysed in 0.2% NP40 in H_2_O for 5 min, scraped from the plates, and transferred into Eppendorf tubes. Lysis was completed by two times tip-sonication for 10 s each. Ex vivo tumors were homogenized by tip sonication. We then extracted 200 μL of the lyates in CHCl_3_:CH_3_OH:1N HCl (100:100:1, v/v/v), centrifuged for 5 min at 14,000 rpm, and collected the lower phase. The samples were dried, resuspended in 20 μL of a detergent solution (7.5% [w/v] n-octyl glucopyranoside, 5 mM cardiolipin in 1 mM diethylenetriaminepentaacetic acid [DTPA]), and sonicated for 10 min to obtain micelles. The kinase reaction was initiated by adding 70 μL of a reaction mixture containing 10 μL diacylglycerol (DAG) kinase (GE Healthcare Europe, Munich, Germany), 0.1 M imidazole/HCl (pH 6.6), 0.2 mM DTPA (pH 6.6), 70 mM NaCl, 17 mM MgCl_2_, 1.4 mM ethylene glycol tetraacetic acid, 2 mM dithiothreitol, 1 μM adenosine triphosphate (ATP), and 5 μCi [^32^P]γATP (6000 Ci/mmol; Hartmann Radiochemicals, Braunschweig, Germany) and performed for 30 min at room temperature with 300 rpm shaking. Kinase assays were terminated by the addition of 1 mL CHCl_3_:CH_3_OH:1N HCl (100:100:1, v/v/v), 170 μL buffered saline solution (135 mM NaCl, 1.5 mM CaCl_2_, 0.5 mM MgCl_2_, 5.6 mM glucose, 10 mM HEPES [pH 7.2]), and 30 μL of a 100-mM EDTA solution. Phases were separated, the lower phase was collected, dried in a SpeedVac, separated on Silica G60 thin-layer chromatography (TLC) plates with chloroform/acetone/ methanol/acetic acid/H_2_O (50:20:15:10:5, v/v/v/v/v), and developed with a Fuji phosphoimager. Ceramide amounts were determined by comparison with a standard curve using C_16_ to C_24_ ceramides as substrates.

### Sphingosine measurements

Cells or tumor tissues were lysed and extracted as for ceramide and finally resuspended in 20 μL of a detergent solution (7.5% [w/v] n-octyl glucopyranoside, 5 mM cardiolipin in 1 mM diethylenetriaminepentaacetic acid [DTPA]) and sonicated for 10 min as above. The kinase reaction was performed for 30 min by addition of 0.001 units sphingosine kinase in 50 mM HEPES (pH 7.4), 250 mM NaCl, 30 mM MgCl_2_ 1 mM ATP and 10 μCi [^32^P]γATP in a volume of 80 μL. Samples were extracted by addition 20 μL 1N HCl, 800 μL CHCl_3_:CH_3_OH:1N HCl (100:200:1, v/v/v), and 240 μL each of CHCl_3_ and 2 M KCl. Samples were vortexed between additions. Phases were separated, the lower phase was collected, dried, dissolved in 20 μL CHCl_3_:CH_3_OH (1:1, v/v), and separated on Silica G60 TLC plates with CHCl_3_:CH_3_OH:acetic acid:H_2_O (90:90:15:5, v/v/v/v) as developing solvent. The TLC plates were analyzed with a phosphorimager. Sphingosine levels were determined with a standard curve of C18-sphingosine.

### Protein measurements

Protein was measured employing the BioRad Protein Assay Dye (cat. no. #500006) from aliquots of cell or tissue lysates. Protein concentrations were used to normalize the samples.

### Ca^2+^ measurement

Cells were loaded with 5 μM Cal520^®^AM (AAT Bioquest) for 60 min at 37 °C, followed by an additional 60 min incubation at room temperature. Cells were washed twice and treated with 1 μM PAPTP, 10 μM FTY-720 or 25 nM Thapsigargin as indicated. Incubation with BAPTA-AM started 30 min prior to PAPTP and FTY-720 treatment.

Fluorescence was measured at the indicated time points with an Elisa-Reader BMG Labtech Fluostar Omega. Changes in fluorescence intensity were normalized to time point 0 using MARS software.

### ATP measurements

ATP was quantified in cell lysates after the indicated treatment by employing a commercial kit following exactly the instructions of the vendor (Abcam # 83355).

### Quantification and statistical analysis

Data are expressed as arithmetic means ± SD. For the comparison of continuous variables from independent groups with one variable (treatment), we used one-way ANOVA followed by post-hoc Tukey test for all pairwise comparisons, applying the Bonferroni correction for multiple testing. The *p* values for the pairwise comparisons were calculated after Bonferroni correction. All values were tested for normal distribution using the Kolmogorov Smirnov test. Statistical significance was set at a *p* value of 0.05 or lower (two-tailed). Outliers were not removed. The sample size planning was based on the results of two-sided Wilcoxon-Mann–Whitney tests (free software: G*Power, Version 3.1.7, University of Duesseldorf, Germany). Based on previous experiments and the literature, we assumed a variation for each experiment and based on this assumption, the n-numbers were calculated using G*Power. In the G*Power program, we used *F*-test, ANOVA (fixed, one-way). The alpha error was set at 0.05, the power at 0.95, and the effect size between 0.5 and 0.9, depending on the assay type, since we assumed that any differences less than 50% would be biologically irrelevant. Investigators were blinded to results of histologic analyses and to animal identity. Animals were randomly assigned to cages by a technician who was not involved in the experiments; thus, the mice were purely randomly assigned for every experiment. Cages were then randomly assigned to the various experimental groups.

## Results

### PAPTP and FTY-720 synergistically kill pancreas cancer cells in vitro and in vivo

**Fig. 1:**
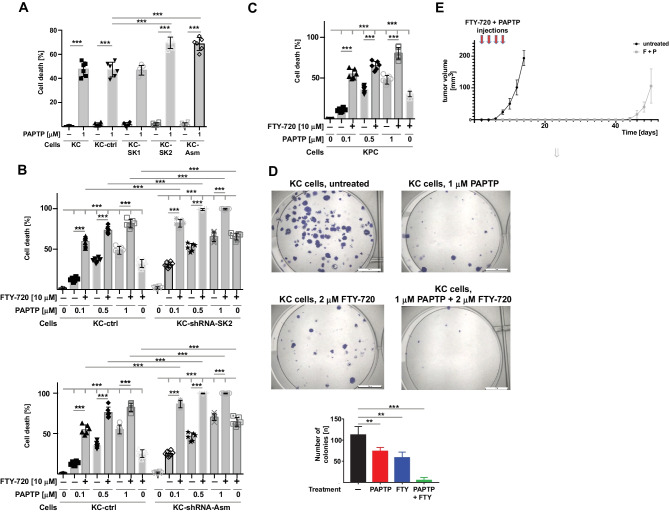
The combination of PAPTP and FTY-720 kills pancreas cancer cells. **A** KC murine pancreas cancer cells were transfected with shRNA targeting sphingosine kinase 1 (KC-SK1), sphingosine kinase 2 (KC-SK2), or acid sphingomyelinase (KC-Asm), with an irrelevant shRNA (KC-ctrl) or left untransfected. Cells were treated with 1 μM PAPTP for 6 h and cell death was determined by FITC-Annexin V staining and flow cytometry. Downregulation of sphingosine kinase 2 or acid sphingomyelinase enhanced the effects of PAPTP on cell death. **B**–**E** The combination of PAPTP with FTY-720 induces up to almost complete cell death in KC (**B**) and KPC cells (**C**). The effects of PAPTP + FTY-720 are enhanced by shRNA-mediated downregulation of sphingosine kinase 2 or acid sphingomyelinase. PAPTP was applied in increasing concentrations from 0.1 to 1 μM; FTY-720 was used at 10 μM. Cell death was determined after 6 h by FITC-Annexin V staining and quantified by flow cytometry. Clonogenic assays (**D**) confirm the synergistic effect of 1 μM PAPTP + 2 μM FTY-720 on cell death in pancreas cancer cells. **E** In vivo studies demonstrate that the combined application of PAPTP + FTY-720 at day 3, 5, 7, and 9 after injection of pancreas cancer cells blocks tumor growth for approximately 40 days, but does not result in complete elimination of the tumor. Shown are the mean ± SD of 6 (**A–C**, **E**) or 3 (**D**) independent experiments or mice or a representative photography from 3 independent experiments. *p < 0.05, **p < 0.01, ***p < 0.001, determined by ANOVA and post hoc Tukey test (**A**, **B**, **C**, **E**) or Dunnett´s multiple comparisons test (**D**)

To define whether the sphingolipid metabolism contributes to the induction of cell death by PAPTP, we transfected pancreas cancer KC cells with shRNA targeting the acid sphingomyelinase, sphingosine kinase 1 or sphingosine kinase 2 (Fig. 1A). We then determined cell death in untransfected cells, control-transfected cells, and in the targeted cells. The results indicate that downregulation of the acid sphingomyelinase or sphingosine kinase 2 increased cell death induced by PAPTP, while downregulation of sphingosine kinase 1 was without effect on cell death (Fig. 1A).

These data indicate an important role of the acid sphingomyelinase and sphingosine kinase 2 for PAPTP-induced cell death. Sphingosine kinase 2 generates sphingosine 1-phosphate. Therefore, we tested whether FTY-720 that is phosphorylated to FTY-720-phosphate and then blocks sphingosine 1-phosphate receptors amplifies cell death induced by different doses of PAPTP. To this end, we determined cell death after treatment of two mouse PDAC lines harboring K-Ras mutation (KC cells or KPC cells) with PAPTP or FTY-720 alone or after incubation with a combination of PAPTP and FTY-720 at different concentrations. The results indicate a marked synergistic effect of 1 μM PAPTP + 10 μM FTY-720 with rapid killing of both pancreas cancer cell lines and 80% death at 6 h after treatment (Fig. 1B, C).

In addition, colony formation in 2D culture and anchorage-independent clonogenic growth in 3D culture were almost completely prevented by the treatment with PAPTP in combination with FTY-720 (Fig. 1D).

Since these studies indicate a very strong induction of tumor cell death and a drastic impairment in colony formation by a combined treatment of the cells with PAPTP and FTY-720, we tested in vivo whether the combination of both drugs has an effect on tumor growth. Tumors were injected into the flank of mice and the mice were treated with 10 mg/kg FTY-720 and 3 mg/kg PAPTP in combination. The results show that 4 injections of PAPTP + FTY-720 results in a marked downregulation and substantial delay of tumor growth (Fig. 1E). However, although we observed a marked growth delay, we did not observe complete elimination of the tumor at the present conditions.

### FTY-720 inhibits acid sphingomyelinase in pancreas cancer cells

Given the promising in vivo result, we decided to gain further insight into the synergistic effect of the two drugs. Surprisingly, the effects of FTY-720 on PAPTP-induced killing or of FTY-720 alone were amplified by downregulation of sphingosine kinase 2 (Fig. 1A, B). Since downregulation of sphingosine kinase 2 should prevent the phosphorylation of FTY-720 and thereby also prevent inhibition of sphingosine 1-phosphate receptors by FTY-720, these data strongly suggest that FTY-720 does not act via inhibition of sphingosine 1-phosphate receptors expressed in the cancer cells.

In addition, downregulation of the acid sphingomyelinase sensitized the tumor cells to PAPTP + FTY-720 or FTY-720-induced cell death and 100% of the tumor cells died already 8 h after treatment, as described above (Fig. 1C). Since downregulation of acid sphingomyelinase promotes cell death induced by PAPTP + FTY-720 or FTY-720 alone, we hypothesized that FTY-720 enhances PAPTP-induced cell death by further inhibiting acid sphingomyelinase.

**Fig. 2:**
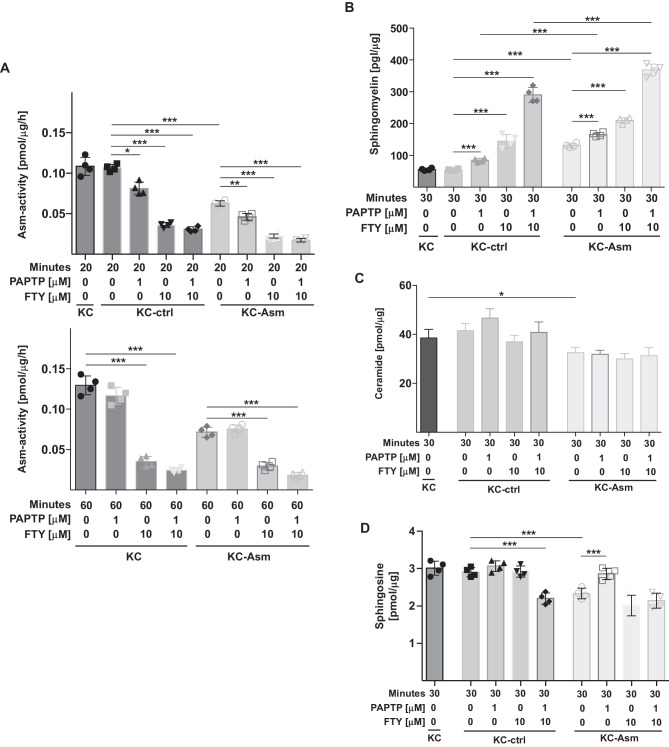
PAPTP + FTY-720 inactivate acid sphingomyelinase (Asm) resulting in increased cellular sphingomyelin. KC cells transfected with control shRNA (ctrl) or shRNA targeting the acid sphingomyelinase (Asm) were treated with PAPTP, FTY-720, or the combination of PAPTP + FTY-720. We then determined the activity of the acid sphingomyelinase 20 min (**A**), the concentration of sphingomyelin after 30 min (**B**), and of ceramide (**C**) or sphingosine (**D**) 30 min after incubation with the drugs. Sphingomyelin was determined by an ELISA (**B**), ceramide and sphingosine were quantified by kinase assays. Shown are the mean ± SD from 6 independent experiments. *p < 0.05, **p < 0.01, ***p < 0.001, determined by ANOVA and post hoc Tukey test

Thus, to test this hypothesis and to gain insight about the mechanism both in vitro and in vivo, we determined the activity of the acid sphingomyelinase in murine KC and KPC pancreas carcinoma cells treated with PAPTP, FTY-720 or the combination of PAPTP + FTY-720. The results show that FTY-720 alone and the combination of PAPTP + FTY-720 induced a marked and rapid inhibition of the acid sphingomyelinase (Fig. 2A). Surprisingly, PAPTP alone also induced some inhibition of the acid sphingomyelinase, likely by an indirect action (Fig. 2A), but this effect was not additive to the effect of FTY-720. PAPTP and FTY-720 also did not act synergistically on acid sphingomyelinase activity.

Transfection of shRNA targeting acid sphingomyelinase resulted in a reduction of acid sphingomyelinase activity by approximately 50% and treatment of the transfectants with FTY-720 or PAPTP + FTY-720 resulted in a further inhibition of the acid sphingomyelinase (Fig. 2A). Control shRNA had no effect on acid sphingomyelinase activity compared to untransfected cells (Fig. 2A). Treatment of the controls with PAPTP + FTY-720 also inhibited the acid sphingomyelinase, but less than in the cells transfected with shRNA targeting acid sphingomyelinase (Fig. 2A).

### The combination of PAPTP and FTY-720 induces an increase of sphingomyelin levels in pancreas cancer cells

Next, we investigated whether inhibition of the acid sphingomyelinase by FTY-720 with concomitant induction of mitochondrial dysfunction by PAPTP treatment has effects on sphingomyelin, ceramide, and sphingosine within tumor cells. The results demonstrate that treatment of KC and KPC cells with the combination of FTY-720 and PAPTP resulted in a marked and very rapid accumulation of sphingomyelin, while FTY-720 or PAPTP alone only moderately affected cellular sphingomyelin concentrations (Fig. 2B). The accumulation of sphingomyelin was most pronounced in the cells transfected with shRNA targeting acid sphingomyelinase, which resulted already in a constitutive increase of sphingomyelin (Fig. 2B).

Ceramide and sphingosine concentrations were not or only slightly affected by PAPTP, FTY-720, or the combination of PAPTP and FTY-720 (Fig. 2C, D).

### PAPTP and FTY-720 inhibit acid sphingomyelinase and induce sphingomyelin in pancreas cancer in vivo

**Fig. 3:**
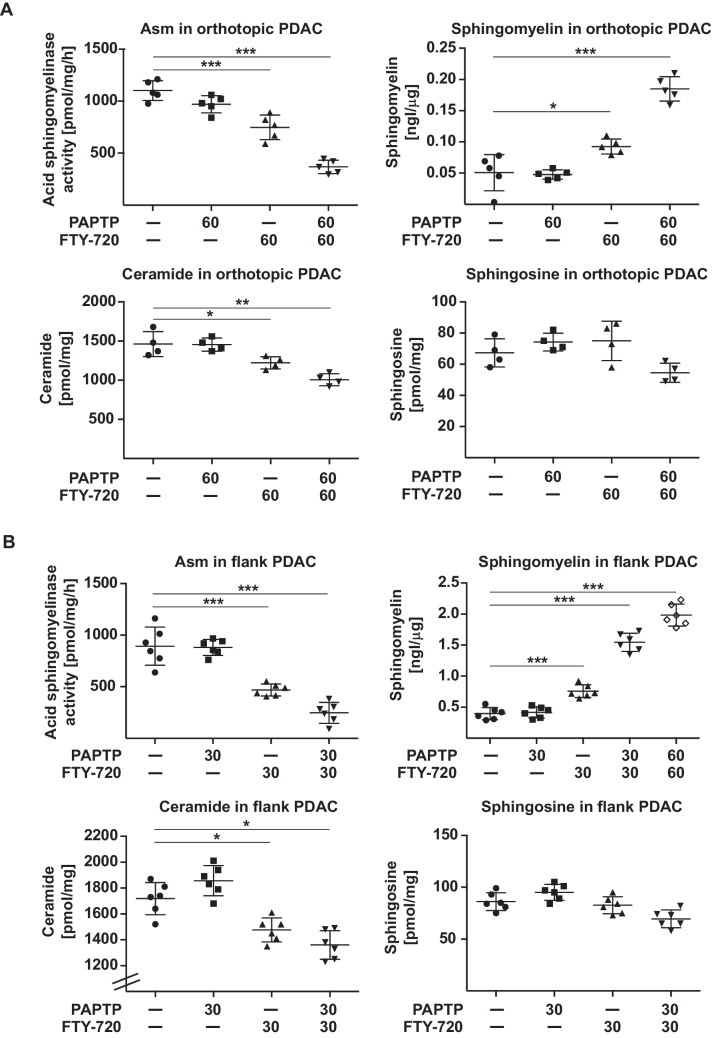
In vivo, PAPTP + FTY-720 inactivates acid sphingomyelinase (Asm) and increases sphingomyelin in pancreas cancer. Mice were injected with 50 000 pancreas cancer KC cells orthotopically into the pancreas (**A**) or into the flank (**B**). The tumor was allowed to grow for 8 day. The mice were then injected with 3 mg/kg PAPTP, 10 mg/kg FTY-720 or the combination of PAPTP and FTY-720. The tumors were removed 30 min or 60 min, respectively, after injection the drugs, lysed and homogenized and the activity of the acid sphingomyelinase and the concentrations of sphingomyelin, ceramide, and sphingosine were determined. Shown are the mean ± SD or representative confocal microscopy studies from 5 (**A**) or 6 (**B**) independent experiments. *p < 0.05, **p < 0.01, ***p < 0.001, determined by ANOVA and post hoc Tukey test

In order to link these in vitro finding to the events taking place in PDAC in vivo upon treatment, we determined the acid sphingomyelinase activity in orthotopic pancreas carcinoma and in pancreas cancer cells injected into the flank of syngenic C57BL/6 mice. The results confirm the data on cultured cells and demonstrate a marked inhibition of the acid sphingomyelinase upon treatment with PAPTP + FTY-720 in orthotopic and flank-injected pancreas cancer (Fig. 3A, B). Similar to the in vitro studies on cultured cells, we also observed a marked increase of sphingomyelin in orthotopic pancreas cancer or flank pancreas cancer samples upon treatment of the mice with PAPTP and FTY-720 (Fig. 3A, B). Ceramide and sphingosine only slightly changed after PAPTP and FTY-720 in vivo (Fig. 3A, B).

### Electron microscopy of pancreas cancer cells reveals massive changes in mitochondria and lysosomes after treatment with PAPTP and FTY-720

**Fig. 4:**
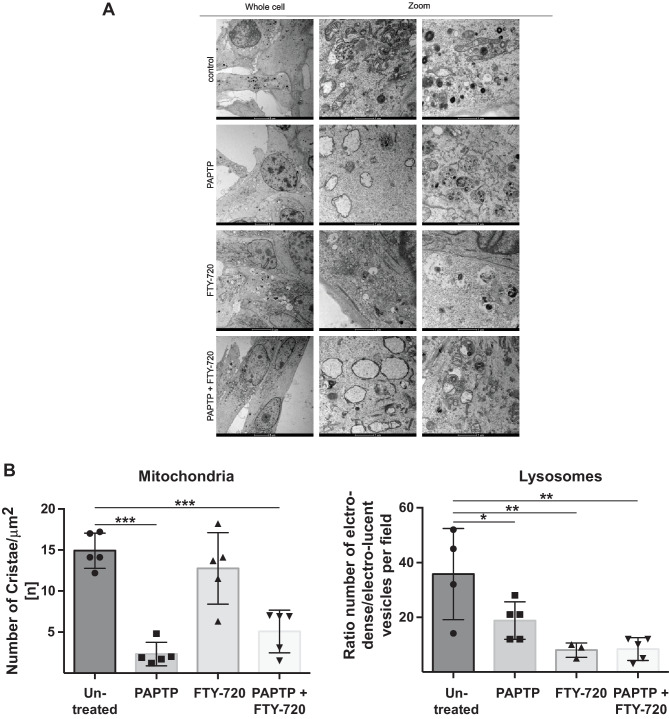
Electron microscopy studies demonstrate targeting of mitochondria and lysosomes by PAPTP + FTY-720. **A** Transmission electron microscope images of KC cells treated for 180 min with 0.1% DMSO (control), 1 μM PAPTP, 10 μM FTY-720 or a combination of both. In control cells, mitochondria show organized cristae structures and numerous electron-dense lysosome sare visible. PAPTP, alone or in combination with FTY-720, leads to swollen mitochondria and disrupted cristae. Additionally, multivesicular bodies are visible. FTY-720 leads to a reduction of electron-dense lysosomes, the appearance of small, electron-lucent vesicles, and autophagosome formation. For each sample, at least 5 individual cells were analyzed. **B** The electron microscopy figures were quantified by counting the number of cristae in mitochondria and the number of electro-dense (functional) vs -lucent (impaired) lysosomes. Shown are the mean ± SD from 3–5 cells. **p < 0.01, ***p < 0.001, determined by ANOVA and post hoc Tukey test

As mentioned above, FTY720 + PAPTP-induced sphingomyelin accumulation in lysosomes might lead to lysosomal membrane permeabilization. To define ultrastructural changes induced by PAPTP, FTY-720, and the combination of PAPTP and FTY-720, we performed electron microscopy. These data indicate that PAPTP disrupts mitochondrial function (Fig. 4A), consistent with previous data [8]. Upon 180 min of PAPTP treatment, mitochondria appear swollen and have lost their cristae structures. In addition, numerous multivesicular bodies are visible in PAPTP-treated cells, indicating increased endo-lysosomal trafficking. FTY-720 alone induced smaller changes in mitochondrial ultrastructure, but as expected, severely affected the lysosomal network: the electron-dense vesicles visible in control cells largely disappeared, while electron-lucent vacuoles and autophagosomes increased in the cytoplasm. The combination of both drugs disrupted mitochondria and lysosomes, leading to mitochondrial swelling, loss of mitochondrial ultrastructure, autophagosome formation, and a reduction of electron-dense vesicles, i.e. lysosomes (Fig. 4A). Shorter treatments (30 min) induced similar, but less pronounced changes in the above-mentioned processes (not shown). Quantification of the number of cristae in mitochondria and the number of electrodense vs lucent vesicles reveals the marked effects of PAPTP + FTY-720 mitochondria and lysosomes (Fig. 4B). The decrease of the number of cristae after PAPTP or PAPTP + FTY-720 indicates a severe impairment of mitochondria, the decrease of the number of electrodense lysosomes after PAPTP, FTY-720, or PAPTP + FTY-720 indicates a severe impairment of lysosomes.

### PAPTP and FTY-720 induce hyperpolarization and reactive oxygen species

**Fig. 5:**
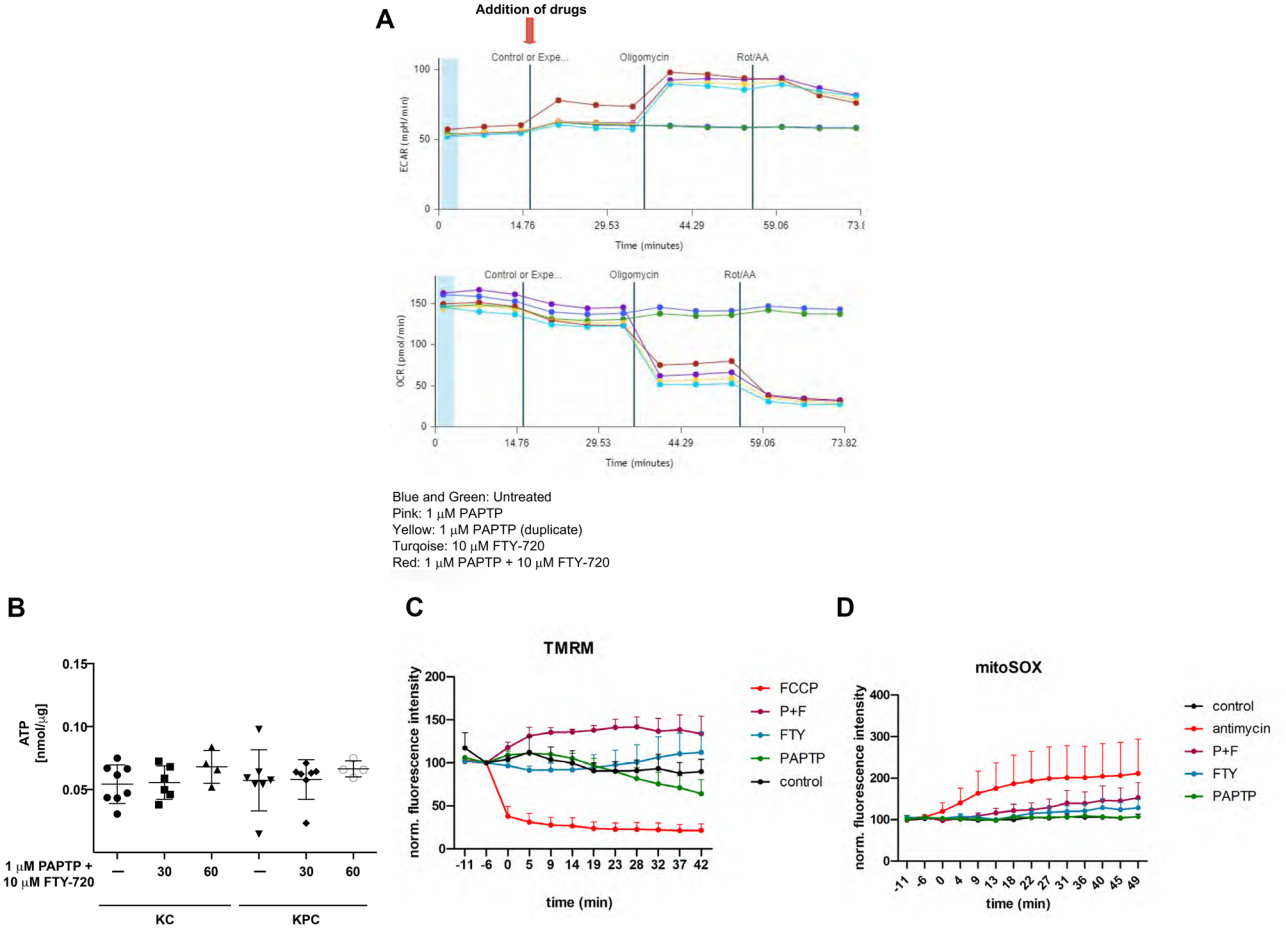
PAPTP + FTY-720 affects mitochondrial functions. **A** KC cells were analyzed by Seahorse prior and after addition of PAPTP, FTY-720, or PAPTP + FTY-720. None of the drugs had a significant impact on basal respiration rate, measured as oxygen consumption rate (OCR), while the drug combination slightly impaired ATP-dependent respiration. Extracellular acidification rate (ECAR) increased after PAPTP + FTY-720 suggesting an increase of glycolytic ATP production upon treatment with PAPTP and FTY-720 in combination. **B** Total cellular ATP concentrations did not change within 60 min after treatment with PAPTP + FTY-720. Shown are representative traces from 6 independent studies (**A**) or the mean ± SD of 6 independent measurements (**B**). *p < 0.05, **p < 0.01, ***p < 0.001, determined by ANOVA and post hoc Tukey test. **C**, **D** Analysis of the mitochondrial membrane potential (**C**) and the formation of mitochondrial reactive oxygen species (**D**) reveal a small increase of the mitochondrial membrane potential after 1 μM PAPTP and 10 μM FTY-720. No change of mitochondrial reactive oxygen species after PAPTP + FTY-720 treatment was recorded by mitoSOX staining. FCCP (1 μM) and antimycin A (5 μM) were used as positive controls for TMRM and mitoSOX, respectively. Shown are the results from three independent experiments

**Fig. 6:**
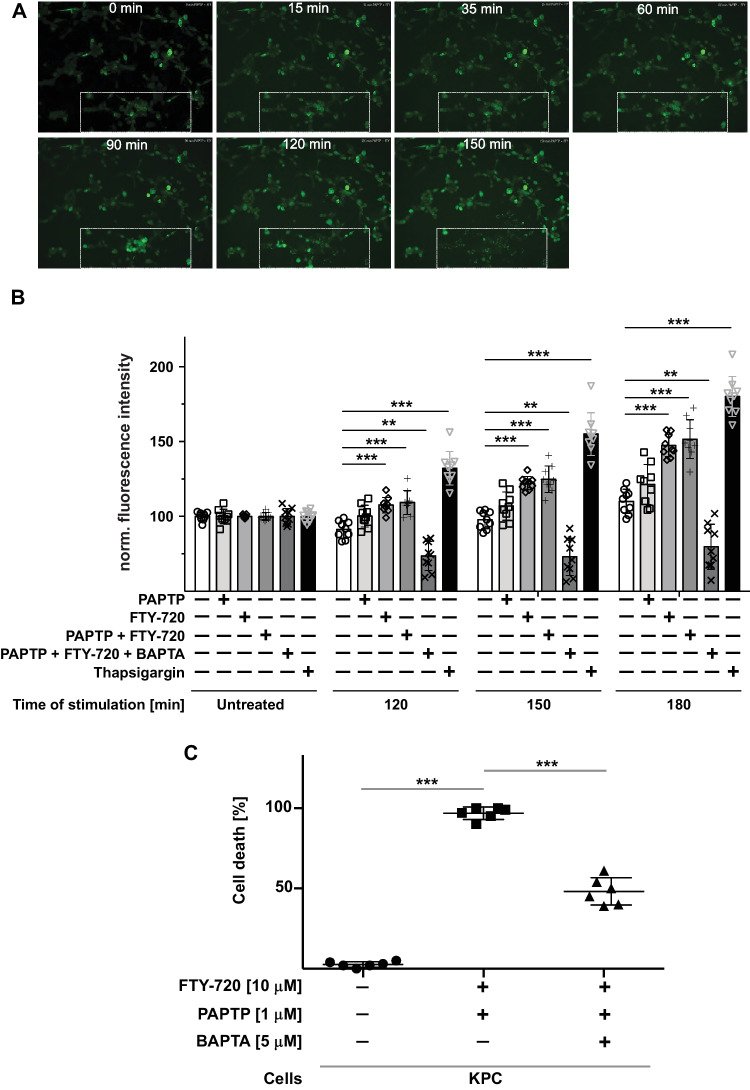
PAPTP and FTY-720 induce massive cytoplasmic Ca^2+^ accumulation and thereby cell death. **A**, **B** KC cells were loaded with 5 μM Cal520^®^-AM to detect cytoplasmic Ca^2+^ and fluorescence was measured 0, 35, 60, 90, 120, 150, and 180 min (**A**) or 0, 120, 150, and 180 min (**B**) after treatment with PAPTP (1 µM), FTY-720 (10 µM), a combination of both ± BAPTA-AM (10 µM) or Thaspigargin (25 nM), employing fluorescence microscopy or a fluorescence reader. Shown are the mean ± SD of the normalized fluorescence intensity of 3 independent experiments, each performed in triplicate, or representative examples from 3 independent studies. *p < 0.05, **p < 0.01, ***p < 0.001, determined by ANOVA and post hoc Tukey test. **C** KPC cells were stimulated with PAPTP and FTY-720 in the presence or absence of 5 μM BAPTA, a Ca^2+^ chelator. Cell death was determined after 24 h in the presence of PAPTP and FTY-720. BAPTA reduced cell death by FITC-Annexin V staining and flow cytometry. Shown are the mean ± SD of dead cells from 6 independent experiments. *p < 0.05, **p < 0.01, ***p < 0.001, determined by ANOVA and post hoc Tukey test

Mitochondria with such a heavily altered ultrastructure are expected to have impaired bioenergetics efficiency. Therefore, we performed extracellular flux analysis with Seahorse, to define mitochondrial respiration upon treatment with PAPTP, FTY-720, and PAPTP + FTY-720. However, none of the drugs had a significant impact on the basal oxygen consumption rate (OCR) (Fig. 5A). In addition, PAPTP + FTY-720, but not FTY-720 or PAPTP alone increased the extracellular acidification rate (ECAR; Fig. 6A). However, total cellular ATP concentrations did not change within 60 min after treatment with PAPTP + FTY-720 (Fig. 5B), consistent with a relatively mild effect of the drugs on mitochondrial bioenergetics, at least after a 20 min stimulation. Incubation with TMRM revealed that PAPTP and FTY-720 induced an increased staining suggesting an apparent hyperpolarization of the mitochondria, which is consistent with the Seahorse studies (Fig. 5C).

### PAPTP and FTY-720 increase cytosolic Ca^2+^ that is involved in cell death

Since both FTY720 and PAPTP have been linked to ROS-mediated death [8, 34], we determined mitochondrial oxygen radical release. The measurements of mitochondrial reactive oxygen species revealed a small, but significant increase of mitochondrial reactive oxygen species after PAPTP + FTY-720 treatment with the low concentrations used in the present study (Fig. 5D).

Mitochondria and lysosomes also serve as Ca^2+^ stores and therefore a simultaneous impairment of mitochondrial and lysosomal functions may result in increased concentrations of cytoplasmic Ca^2+^, which may then induce cell death. Indeed, cytoplasmic Ca^2^ increased in KC cells after treatment with PAPTP + FTY-720 over time (Fig. 6A, B). To test whether the increase of cytoplasmic Ca^2+^ contributes to cell death, we incubated KPC cells with 5 μM BAPTA, a Ca^2+^ chelator, in the presence of PAPTP and FTY-720. BAPTA reduced cytoplasmic Ca^2+^ (Fig. 6B) and cell death (Fig. 6C) by approximately 50% indicating that Ca^2+^ contributes to cell death.

## Discussion

In the present study we demonstrate a marked synergistic effect between mitochondrial and lysosomal dysfunction inducing massive death of PDAC cells. We employed the mitochondrial Kv1.3 blocker PAPTP and the drug FTY-720 to study their effects on death of pancreas cancer cells in vitro and also in vivo. FTY720 was shown to reduce proliferation of PDAC cells [25] and has been reported to enhance non-canonical cell death in PDAC cells when applied together with the epidermal growth factor receptor/HER2 inhibitor lapatinib [24]. Furthermore, FTY-720 induced necrotic death of ovarian cancer cells [26]. However, the in vivo effects of FTY720 either alone or in combination against PDAC have not been tested.

Here, we demonstrate that FTY-720 targets lysosomes inducing an accumulation of sphingomyelin by inhibition of the acid sphingomyelinase. High lysosomal sphingomyelin levels might contribute to lysosomal membrane permeabilization and thereby cell death [18, 19]. In accordance, FTY-720 has recently been reported to induce lysosomal membrane permeabilization in glioblastoma cells [33] and in PDAC in combination with lapatinib [24]. Ca^2+^ sequestered in lysosomes may leak following lysosomal membrane permeabilization, which may affect Ca^2+^ regulation in the endoplasmatic reticulum (ER) and mitochondria, resulting in ER stress and mitochondrial dysfunction. In addition, FTY-720 also has some mitochondrial effects as evidenced by the electron microscopy studies. The mitochondrial effect of FTY-720 might be mediated by targeting mitochondrial acid sphingomyelinase, although this needs to be further investigated. We observed a higher accumulation of sphingomyelin after the combination of both drugs than after single application, although the drugs did not induce a synergistic inhibition of the acid sphingomyelinase. This might be explained by additional effects of the combination of the two drugs on sphingomyelin synthesis and/or an additional inhibition of neutral sphingomyelinases; however, this finding requires further detailed studies on sphingomyelin metabolism.

Ceramide is not only generated by the activity of the acid sphingomyelinase, but also via the ceramide synthase pathway and by neutral sphingomyelinases. Thus, the inhibition of the acid sphingomyelinase may not result in a marked decrease of ceramide, since other pathways may balance the reduced formation of ceramide. Likewise, the levels of sphingosine are not entirely controlled by the activity of the acid sphingomyelinase, consistent with less pronounced changes in sphingosine levels after PAPTP and FTY-720.

Previous studies suggested that FTY-720 induces a degradation of the acid sphingomyelinase [22], but FTY-720 does not fulfill the structural requirements to displace the acid sphingomyelinase from the membrane, which is a positively charged head group that is linked to a hydrophobic part via a relatively long and flexible linker [27] and the mechanisms how FTY-720 and also PAPTP reduce acid sphingomyelinase activity remains to determined in future studies.

The effect of PAPTP on mitochondrial acidification might be indirectly mediated by a change of mitochondrial energy metabolism and the release of reactive oxygen species upon inhibition of mitochondrial Kv1.3. A communication between mitochondria and lysosomes has been recently shown, although these data investigated long-term effects of mitochondrial changes on lysosomal functions [28]. However, it might be possible that an acute change of the mitochondrial membrane potential and mitochondrial metabolism affects lysosomal membranes with the result of an increased permeability and/or inhibition of the interaction of lysosomal membranes with associating proteins such as the acid sphingomyelinase. Such an alteration of the binding properties of the lysosomal membrane to proteins would result in a release of the acid sphingomyelinase into the lysosomal lumen and thereby degradation of the protein.

Thus, we propose the following mechanism of action: FTY-720 blocks the acid sphingomyelinase resulting in massive accumulation of sphingomyelin. PAPTP inhibits mitochondrial Kv1.3 resulting in changes of the mitochondrial membrane potential, ROS production, and mitochondrial metabolism. The accumulation of sphingomyelin, together with signals from the impaired mitochondria (such as ROS), disrupts lysosomal integrity, further promoting acid sphingomyelinase inhibition and sphingomyelin accumulation. The dysfunction of lysosomes and mitochondria impairs storage of Ca^2+^ in these organelles resulting in high Ca^2+^ concentrations within the cytoplasm and thereby cell death. In addition, FTY-720 may have direct effects on mitochondria that act synergistically with PAPTP. On the other hand, it has been shown that mitochondrial ROS was shown to activate efflux of Ca^2+^ from lysosomes through the ROS-sensitive ion channel TRPML1 [29]. Thus, mitochondrial ROS might contribute to Ca^2+^ release also in our system, although this mechanism is less likely since sphingomyelin inhibits TRPML1 activity [30].

FTY-720 is a pro-drug that is phosphorylated by sphingosine kinases and then binds to sphingosine 1-phosphate receptors and blocks these receptors. It is unlikely that FTY-720 kills the tumor cells via this mechanism, since PAPTP and FTY-720 induced cell death very rapidly. Furthermore, transfection of cells with shRNA downregulating sphingosine kinase 2 increased the effects of FTY-720 on pancreas cancer cell death, which is consistent with a direct effect of FTY-720, but inconsistent with an effect of phosphorylated FTY-720. However, it is possible that sphingosine 1-phosphate protects mitochondria from the effects induced by PAPTP and that blockade of this protective effect results in massive changes in mitochondria that then also change lysosomal functions. However, since we did not observe any change of cellular ATP, only very modest changes of succinate (not shown) and no severe changes in mitochondrial oxygen consumption immediately after PAPTP and FTY-720, such a scenario seems to be very unlikely.

We demonstrate that cellular treatment with PAPTP + FTY-720 results in a rapid and massive release of Ca^2+^ and cell death after PAPTP and FTY-720 is partially prevented by treatment with the intracellular Ca^2+^ chelator BAPTA. This suggests that the increased concentrations of cytoplasmic Ca^2+^ observed after PAPTP and FTY-720 significantly contribute to killing the pancreas cancer cells. Lysosomes and mitochondria are known as cellular Ca^2+^ stores [31] and the simultaneous inhibition of their functional integrity by PAPTP and FTY-720 may result in the observed rapid and massive accumulation of Ca^2+^ in the cells after treatment. We have previously shown that treatment with PAPTP triggers opening of the permeability transition pore that is implicated in Ca^2+^ release from damaged mitochondria [32]. A massive increase of cytoplasmic Ca^2+^ has been previously shown by many studies to mediate cell death [33, 34] and we propose that this is also one mechanism how combined application of FTY-720 + PAPTP kills cells. However, the combined impairment of mitochondria and lysosomes may also result in other mechanisms that mediate cell death, for instance reduced or blocked autophagy, which might further contribute to the induction of death.

Our in vivo data show that the combined treatment with FTY-720 and PAPTP greatly delayed tumor growth and extended the time until a similar size of the tumor has been reached by approximately fourfold. This is remarkable for pancreas cancer, since this cancer belongs to the tumors that are very hard to treat. However, we were unable to achieve a complete eradication of the tumor with the treatment. The final failure might be caused by the development of resistance mechanisms, especially in the case of cancer stem cells. It might be possible that an up-regulation of sphingosine-kinase 2 results in increased phosphorylation of FTY-720 and thereby de-toxification in tumor cells in vivo after treatment. In future studies, it might be worthwhile to combine FTY-720 + PAPTP with inhibitors of sphingosine kinase 2. However, we also observed a substantial transient weight loss of the mice and it remains to be determined whether co-application of further drugs results in even stronger toxicity, whether the doses have to be adapted and whether this has an impact on the treatment.

In summary, we demonstrate that application of FTY-720 and PAPTP results in rapid and almost complete death of pancreas cancer cells, even in clonogenic assays. We demonstrate that the drugs induce an inhibition of the acid sphingomyelinase, an accumulation of sphingomyelin, and a severe impairment of lysosomal functions, which together with the impairment of mitochondrial functions induced by PAPTP prevents storage of Ca^2+^ in these organelles and thereby induces cell death. These data also open a new avenue in the emerging field of mitochondria-lysosome cross-talk.

## Data Availability

All data are presented in the manuscript. All material is freeky available.
